# Extraction and derivatisation of active polysaccharides

**DOI:** 10.1080/14756366.2019.1660654

**Published:** 2019-09-20

**Authors:** Yang Liu, Gangliang Huang

**Affiliations:** Active Carbohydrate Research Institute, Chongqing Key Laboratory of Inorganic Functional Materials, College of Chemistry, Chongqing Normal University, Chongqing, China

**Keywords:** Chinese medicine polysaccharide, extraction, derivatisation

## Abstract

Polysaccharide, as one of the main components of traditional Chinese medicine, has an important antioxidant effect, and its mechanism has been studied by more and more researchers. The preparation methods of Chinese traditional medicine polysaccharides and their derivatives were reviewed. In addition, the advantages and disadvantages of various preparation methods of different Chinese medicine polysaccharides and their derivatives were discussed.

## 1. Introduction

Traditional Chinese medicine originated in China has a unique theoretical system. It is mainly used for the prevention and treatment of diseases. Traditional Chinese medicine polysaccharide is one of the effective components of many traditional Chinese medicines and has many functions such as anti-oxidation. In addition, the extraction, purification, and mechanism of Chinese herbal polysaccharides are also concerns of many researchers^1^.

The traditional extraction method of Chinese traditional medicine polysaccharide is water-boiling method. It is very convenient, but also has many disadvantages, such as many ingredients dissolve in high temperature, some active ingredients will deteriorate, which may reduce the efficacy of traditional Chinese medicine. In order to extract polysaccharides from traditional Chinese medicine, traditional Chinese medicine contains a lot of polysaccharides. Another method is to boil the ethanol and precipitate it. In recent years, some researchers have adopted other methods, such as microwave-assisted extraction, hydroenzyme-assisted extraction, vacuum extraction, ultrasonic-assisted extraction, etc., to extract polysaccharides more rapidly and effectively.

Antioxidant properties include antioxidant *in vivo* and antioxidant *in vitro*. *In vitro*, the antioxidant activities were mainly hydroxyl radical scavenging, superoxide anion scavenging, reducing power, anti-lipid peroxidation, etc., and the antioxidant capacity *in vivo* was mainly determined by the total antioxidant capacity, superoxide dismutase, and glutathione peroxidase activities. Mouse serum, liver, heart and kidney. At present, the effect of polysaccharides on antioxidant activity is only to verify their antioxidant properties, but the relationship between antioxidant activity and structure is not clear. In order to clarify the structure and oxidation mechanism of polysaccharides, this paper mainly reviews the effect of polysaccharides on antioxidant activity. Extraction method of Chinese traditional medicine polysaccharide, antioxidant activity *in vivo* and *in vitro*, structure and composition of main monosaccharides.

## 2. Extraction of polysaccharide from traditional Chinese medicine

### 2.1. Water–alcohol precipitation

The process route is as follows: traditional Chinese medicine → water frying, filtration → concentration → ethanol precipitation → overnight low temperature → centrifugation → washing and drying → crude polysaccharide^2^. The following are the extraction methods and conditions of several kinds of traditional Chinese medicine polysaccharides.

In order to extract polysaccharides from golden lotus, we used three different methods to extract polysaccharides from golden lotus. The extraction rate of polysaccharide by conventional reflux method was 1.21%, that by ultrasonic method was 0.24%, and that by supercritical method was 0.00187%. Therefore, the best method to extract polysaccharide is conventional reflux method. Through single-factor test and orthogonal test, the optimum technological conditions for extraction of jinlian polysaccharide by traditional reflux method were determined as follows: water was 20 times that of jinlian polysaccharide, and extraction temperature was 95 °C, reflux time was 2 t. For IMES, the extraction rate of polysaccharide was 1.45% for each 3 h period^3^.

Extraction of polysaccharide from atractylodes, cutting of atractylodes, reflux of 75% ethanol for 2 h at 80 °C, boiling three times in the pot, collection of boiling liquid, filtration, ethanol precipitation, ether dehydration, vacuum drying to obtain atractylodes. Big head polysaccharide (mainly composed of galactose, rhamnose, xylose, and arabinose)^4^.

*Extract glycyrrhiza polysaccharide*. The dried glycyrrhizae were extracted with 95% ethanol at 100 °C for three times, respectively, for 2 h, 1.5 h and 1 h. Filtrate is removed by filtration. Filtrate was extracted with hot water for 3 h, 2 h and 2 h, respectively, precipitated with 80% ethanol, placed at 4 °C for 24 h, filtered, washed and dried. Protein was removed by Sevag method, and crude polysaccharide was obtained by distilled water dialysis^5^.

The extraction process parameters of total polysaccharide were studied by orthogonal test, and the effects of water extraction, acid extraction, alkali extraction, and enzymatic extraction of total polysaccharide were compared. The technological parameters of alcohol precipitation were studied by orthogonal test. The methods of removing protein by trichloroacetic acid, hydrochloric acid, and papain were compared. Concentrate the extraction solution, precipitate with ethanol, remove the solution by centrifugation, wash the precipitation with anhydrous ethanol, acetone and anhydrous ether, dissolve with water, add trichloroacetic acid to the solution to a certain volume fraction, and conduct static electricity. City was transferred to neutral state and centrifuged. Supernatant was taken and activated carbon was added for adsorption, centrifugation, concentration, and dialysis^6^.

Mulberry polysaccharide was extracted from mulberry by water extraction and alcohol precipitation method. The optimal extraction conditions were as follows: add water 20 times, ultrasound at 50 °C for 30 min, and extract twice^7^.

### 2.2. Dilute alkali leaching

The process route is as follows: traditional Chinese medicine → low temperature → acid neutralisation → filtrate → alcohol precipitation → low temperature → standing → centrifugation → washing, and drying → crude polysaccharide products^8^.

After pre-treatment, 50 mol/L sodium hydroxide was used to extract xanthate with 1:9 solid–liquid ratio. After stirring at room temperature for 105 min, the residue was repeatedly extracted at 6000 RPM for 20 min. When the clarification solution was mixed, the free protein in the supernatant was removed by ethanol, and the concentration was reduced to reduced pressure. 95% ethanol was added to the 60% ethanol concentration, and the dissolved polysaccharide was allowed to stand for 20 min after 6000 RPM/separation^9^.

### 2.3. Fermentation alcohol precipitation method

Method intracellular polysaccharide extraction and extracellular polysaccharide extraction were used. Extraction process of intracellular polysaccharide^10^ fermentation broth collection of mycelia → distilled water → measurement of wet and dry weight of mycelia → weight extraction by dehumidification hot water → centrifugation collection of supernatant → concentration to acetoacetic acid NOL precipitation → organic solvent low-temperature washing → intracellular polysaccharide. The above steps are used to extract water-soluble polysaccharide from cells, extract-soluble polysaccharide from mycelium through thin alkali extraction mycelium filter, extraction process, and dilute alkali extraction method. Extracellular polysaccharide extraction process is as follows: mycelium collection by fermentation broth centrifugation → concentration → ethanol precipitation → organic solvent washing → low-temperature drying → extracellular polysaccharide.

### 2.4. Enzymatic method

The process route is as follows: traditional Chinese medicine → soak in water → add complex enzyme → add water → add water to heat → continuous extraction → ethanol precipitation → organic solvent washing → crude polysaccharide.

The best extraction method of jujube polysaccharide was as follows: the enzymatic hydrolysis temperature was 56–65 °C, the digestion time was 5 0 ∼ 68 min, the pH value was 5.6–6.3, and the ratio of liquid to material was 20:1. Compared with traditional water bath extraction method. This method not only shortened the extraction time, but also increased the yield of jujube polysaccharide from 8.19% to 12.33%^11^.

### 2.5. Microwave extraction

The best method of microwave extraction of acanthopanax polysaccharides was as follows: the microwave radiation time was 20.8 min, and the drug liquid ratio was 1:22.1 at 100.9 °C. In this case, the extraction rate can reach (5.04 + 0.09)%^12^.

In order to obtain an effective extraction method of polysaccharides from red peony root, microwave-assisted extraction method was used to extract polysaccharides from red peony root. The optimal extraction parameters are as follows: extraction time: 45 min, microwave power: 213 watts, temperature: 80 °C, solid–liquid ratio: 26:1, and extraction time: 1 time. Under these conditions, the content of polysaccharides in red peony root could reach (10.11 ± 0.52)%^13^. The optimal conditions for microwave-assisted extraction of *Bletilla striata* polysaccharide were as follows: the ratio of liquid to material was 75:1, the extraction temperature was 50 °C, the extraction time was 15 min, the microwave power was 700 watts, and the extraction times were two times^14^. The extraction rate of *B. bletilla* polysaccharide was 25.80%, which was better than ultrasonic extraction rate of 13.52%. The main order of influencing factors is as follows: extraction time > liquid–solid ratio > extraction time > extraction temperature^15^.

### 2.6. Ultrasonic extraction

In order to reveal the characteristics of “three treasures of guizhou”, traditional Chinese medicine polysaccharide is more suitable for folk medicinal food. According to the analysis of polysaccharide concentration and response surface of guizhou sanbao, the ratio of substance to liquid is 1:60. The ultrasonic power was 178.23 W, the extraction temperature was 64.93 °C, the ultrasonic time was 26.72 min, and the polysaccharide content was the highest, reaching 26.4%^16^. Bulrush polysaccharides were extracted by hot water extraction, cellulase-assisted hot water extraction and ultrasonic-assisted hot water extraction. The third method has the highest yield, reaching 1.75%^17^.

### 2.7. Ultrasonic assisted enzyme extraction method

The extraction conditions, temperature, time, solid–liquid ratio, and ultrasonic intensity of *Achyrantha bidentata* polysaccharide were optimised by ultrasonic-assisted enzyme method. Experimental data were processed and analysed by software. The optimal extraction conditions were as follows: temperature of 60 °C, extraction time of 30 min, liquid/material ratio of 35 ml/g, and ultrasonic intensity of 300 W^18^.

The polysaccharides were extracted by hot water and ultrasonic wave, respectively. The results showed that the polysaccharide content, extraction rate, antioxidant activity, and antidiabetic activity were better than warm water. Through research, ultrasonic extraction, as a new active component in plant extraction, has great advantages over traditional extraction methods, providing theoretical basis for subsequent ultrasonic extraction methods. The significance of extracting active ingredients of traditional Chinese medicine^19^.

With angelica as raw material, ultrasonic-assisted extraction. The factors and levels were determined by single factor and orthogonal test, and were based on the results of single factor and orthogonal test. Determine their impact on extraction rate: the ratio of liquid to solid to > ultrasonic extraction time > ultrasonic extraction power^20^.

### 2.8. Vacuum extraction

Vacuum extraction technology is a new extraction technology, which combines the vacuum system on traditional extractors to make the solvent boil at a lower temperature to avoid damage to thermal elements. Methods of decompression, hot reflux extraction, ultrasonic extraction, and low-temperature extraction were used to extract salvia miltiorrhiza. The contents of salvianolic acid B, protein, soluble sugar, light transmittance, dry paste, and DPPH free radical scavenging were analysed. The optimal decompression conditions of the extract of salvia miltiorrhiza were optimised., liquid–material ratio 10:1, vacuum degree –0.07 MPa, extraction temperature 50 °C, extraction time 1.0 h^20^.

The optimum extraction conditions of large leaf and grass were the same as that of salvia miltiorrhiza. The extraction rate of resveratrol was slightly higher than that of vacuum extraction, ultrasonic extraction, heated reflux extraction and low-temperature extraction, light transmittance, protein content, soluble sugar content, and DPPH free radical scavenging rate.

*Low-temperature leaching*. However, the extract contains little protein and impurities, good water solubility, low viscosity of polysaccharide, and easy filtration, which is conducive to subsequent processing and suitable for extraction. The effects of curcumin, methoxycurcumin, and dimethoxycurcumin on the extraction rate of three active components were studied by vacuum extraction solvent, liquid–liquid ratio, and extraction time. The optimal extraction conditions of the vacuum method were as follows: 70% ethanol was extracted, the liquid–liquid ratio was 15:1, the vacuum degree was –0.07mpa, the extraction temperature was 50 °C, and the extraction time was 1.5 h. Under these conditions, the total extraction amount was 1.5 h. The extraction rate of curcumin was 3.48%. Compared with traditional extraction methods, the contents of extract, protein, soluble sugar, and DPPH free radical scavenging were determined. The results showed that the yield of curcumin derivatives and the antioxidant capacity of vacuum extraction were higher than other extraction methods. The contents of soluble sugar and protein in vacuum extraction, ultrasonic extraction, and low-temperature extraction showed little difference, but were lower than those in heated reflux extraction. The content of active ingredients in turmeric increased, and impurities such as protein and soluble sugar decreased, indicating that the decompression extraction effect of turmeric was better than other extraction methods.

### 2.9. Pulsed electric field extraction

High-voltage pulsed electric field (PEF) and ultrasonic extraction were used to extract water-soluble polysaccharide from antler. The best extraction method of antler is as follows: the ratio of liquid to material is 12:1, the electric field intensity is 25 kV/cm, the pulse number is 8, and the percentage of papain is 3%^21^.

## 3. Derivatisation of polysaccharides

The activity of polysaccharides is closely related to the structure of polysaccharides. The activity of polysaccharides modified by structure can be significantly improved in some aspects. Therefore, the structural modification of polysaccharides has gradually become a research hotspot. Among them, the chemical modification of polysaccharides is one of the research focuses. Through further research, the following chemical modification methods are obtained: sulphation, phosphorylation, carboxymethylation, acetylation, selenification, alkylation, bi-group derivatisation, etc.

**Figure 1.**
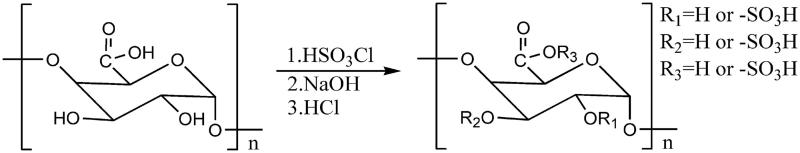
Sulphation of polysaccharides.

Current research shows that the most commonly used experimental methods for sulphation modification of polysaccharides are concentrated sulphuric acid method, SO_3_-pyridine method, HSO_3_Cl-pyridine method, NH_2_SO_3_H-formamide method, SO_3_-DMF method, and HSO_3_Cl-DMF method. Among these, HSO_3_Cl-pyridine, concentrated sulphuric acid, and SO_3_-pyridine are three commonly used methods (Figure 1).

*Chlorosulfonic acid-pyridine method*. Chlorosulfonic acid is a very acidic acid. It will decompose violently when it meets water, and it will emit a lot of heat to carbonise polysaccharides. Therefore, the reagent bottle of the reaction must be kept dry, and water cannot be used as a solvent. Therefore, pyridine is generally used as a medium, and ice salt bath cooling is used to prevent excessive temperature. The reaction is relatively simple and rapid. Therefore, attention should be paid to the proportion of reagents, reaction time, and reaction temperature, which will affect the final yield of sulphated polysaccharides. Therefore, the selection of the optimal conditions for this method is also a research hotspot. Orthogonal experiments or response surface experiments are usually used to study the optimal reaction conditions. For example, Wang et al.^22^ used orthogonal experiment to study various factors (reagent ratio, reaction time, and experimental temperature) that influence the sulphation of SC-FUC (trepang fucoidan sulphate) by HSO_3_Cl-pyridine method, and discussed the optimal experimental scheme of sulphation: the volume ratio of chlorosulfonic acid to pyridine is 1:1 and reaction time is 2. The reaction temperature is 50 C. The content of sulphuric acid group increased from 10.75% to 59.53%. The Fourier transform infrared spectroscopy results show that the intensity of characteristic absorption peaks of sulphated SC-FUC increases obviously.

*Concentrated sulphuric acid method*. The method uses concentrated sulphuric acid as the source of sulphuric acid group, pyridine as the medium, and then chemical modification of polysaccharide residues. The advantages of this method are less toxicity and relatively stable conditions, while the disadvantage is that exothermic conditions can also exist in concentrated sulphuric acid reaction, which can carbonise polysaccharides. For example, Luan et al.^23^ used concentrated sulphuric acid method to sulphate polysaccharides from *Schisandra chinensis* leaves. The best experimental conditions were summarised by single-factor experiments: the reaction temperature and reaction time of 1.4 h at 3:1 and 0 °C. Finally, the substitution degree of sulphate in polysaccharide can reach 0.4597.

*Sulphur trioxide-pyridine method*. The reaction conditions are mild and the properties of the reagents used are stable, but the substitution degree and recovery of sulphate are not very high. For example, Xiao et al.^24^ studied the factors affecting the reaction of SO_3_-pyridine method and *Codonopsis pilosula* polysaccharide (CJP) (experimental time, ambient temperature, and reagent ratio). The optimum reaction conditions were obtained as follows: the ratio of SO_3_-pyridine to polysaccharide solution was 4:1 (m/m), the experimental temperature at 80 °C, and the experimental time of 4 h. The final degree of substitution can be as high as 1.66%. Infrared spectra also show that the sulphation reaction of CJP is successful.

In addition, Zhang et al.^25^ sulphated *Ganoderma lucidum* extracellular polysaccharide with DMF as solvent and aminosulfonic acid as esterification reagent. The degree of substitution of sulphate radical was up to 2.67 when urea was used as catalyst and microwave-assisted reaction was carried out.

**Figure 2.**
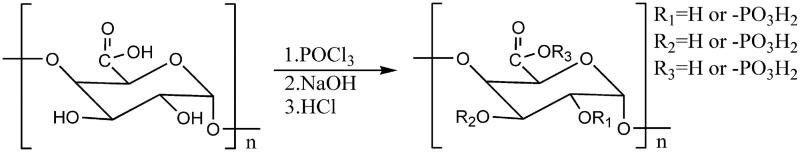
Polysaccharide phosphorylation.

Natural phosphorylated polysaccharides are mostly derived from fungi, while reports from other sources are relatively rare. Therefore, artificial phosphorylation of polysaccharides is not only helpful to the study of phosphorylated polysaccharides, but also can enhance the biological activity of some natural polysaccharides through reaction. At present, phosphoric acid esterification reagents are widely used, such as phosphoric acid, acid anhydride of phosphoric acid, some phosphates, and phosphorus oxychloride (Figure 2).

*Phosphates*. Phosphates for polysaccharide phosphorylation include Na_5_P_3_O_10_, Na_3_ (PO_3_) _3_, Na_2_HPO_4_, and NaH_2_PO_4_. Zhao et al.^26^ studied the phosphate esterification of *Tussilago fargesii* by mixed phosphate method, and studied its physicochemical properties. The experimental conditions were optimised by single-factor experiments. The results showed that the mass ratio of mixed phosphate to polysaccharide was 1.5:1, the mass ratio of Na_2_HPO_4_ to NaH_2_PO_4_ was 2:1, the reaction time was 6 h, and the reaction temperature was 86 °C. Under these experimental conditions, the phosphorylated polysaccharide of *Fagopyrum fargesii* with an average degree of substitution of 0.697 can be obtained.

*Phosphorus oxychloride*. It is also a common phosphorylation reagent. Pyridine is usually used as the medium in the reaction, and the dosage of phosphorus oxychloride should be controlled to prevent the degradation of polysaccharides. For example, Zhang et al.^27^ phosphorylated pectin with phosphorus oxychloride method, optimised the experimental conditions, and tested the immune activity of the phosphorylated apple pectin. The optimum experimental results are as follows: the experimental temperature is 50 °C, the time is 3 h, and the liquid-to-material ratio is 3:1. Under the optimal experimental scheme, the content of phosphoric acid radical reached 11.52%. However, phosphoric pectin with medium phosphorus content had better inhibitory activity than other contents.

**Figure 3.**
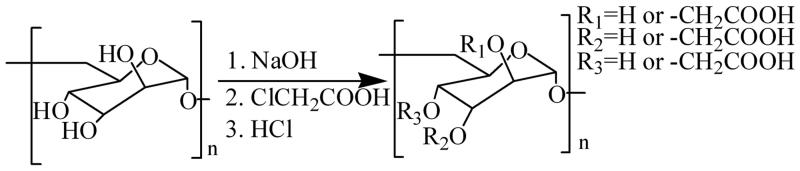
Carboxymethylation of polysaccharides.

By introducing carboxymethyl groups, the water solubility and electronegativity of some polysaccharides were improved, and the antitumor, hypoglycaemic, and antioxidant activities of polysaccharides were further enhanced (Figure 3). Therefore, carboxymethyl reaction of polysaccharides is also of great value. Cao et al.^28^ reacted in NaOH–isopropanol–sodium chloroacetate system to study carboxymethylated phenylglycerol polysaccharide (CM-EPS). Then the activity of CM-EPS was evaluated by scavenging free radicals and NO_2_- and inhibiting the proliferation and apoptosis of HepG-2 cells. Infrared spectrum analysis showed that carboxymethylation modification was successful and the degree of substitution reached 0.961. Compared with natural EPS, the scavenging effect of CM-EPS on ABTS and DPPH radicals was weakened. However, the inhibition of HepG-2 proliferation by CM-EPS was significantly enhanced, and the removal of NO_2_-by CM-EPS was also significantly enhanced.

Natural polysaccharides are macromolecule compounds with many hydroxyl groups. Under suitable chemical reaction conditions, some active hydroxyl groups on the polysaccharide ring can produce corresponding esters through nucleophilic substitution reaction^29^. Xu et al.^30^ *Hericium erinaceus* polysaccharide (Hep) was obtained by hot water extraction and acetylated *Hericium erinaceus* polysaccharide (A-Hep) was prepared. By combining single factor and response surface methodology, the optimum experimental scheme was determined as follows: liquid-to-material ratio was 34:1 (mL/g), reaction time was 3 h, and experimental temperature was 30 °C. Under this experimental condition, the degree of substitution of A-Hep was as high as 0.609, and the antioxidant activity of A-Hep was significantly improved compared with that of non-acetylated HEP.

Selenium is a trace element, which is indispensable to life. It can activate the immune system, exert immune function, and also has the ability to defend against cancer. Polysaccharide is also a natural active substance. Selenium and polysaccharides are combined by chemical reaction to form a selenium polysaccharide with low side effects, low toxicity, and easy absorption, which further shows that the polysaccharides have wider biological activities. Therefore, the study of selenium polysaccharides has important medical significance and experimental value. In Chen et al.^31^, PCPs were synthesised by nitric acid-sodium selenite method. The reaction conditions of PCPs on human lung adenocarcinoma cell line (A549) were optimised by single factor and L_9_ (3^4^) orthogonal test. The effect of PCPs on human lung adenocarcinoma cell line (A549) was also tested. The optimised selenification reaction conditions were as follows: experimental time was 5 h, experimental temperature was 60 °C, and the mass ratio of polysaccharide to Na_2_SeO_3_ was 1:1 (m/m). Under the optimised conditions, the content of selenium in the selenified polysaccharides of *Codonopsis pilosula* can reach 1.07 mg/g (RSD 3.7%); the yield can reach 50.3% (RSD 2.5%), and the inhibition rate to A549 cells can reach 49.36% (RSD 2.8%). Therefore, PCPs can be used as a promising candidate for anti-cancer drugs.

In addition to the above methods, there are other methods for polysaccharide derivatisation, such as alkylation^32^, sulfonylation^33^, and biradical derivatisation, to improve the bioactivity of polysaccharides. A large number of experiments show that the activity of polysaccharides in some aspects will be significantly enhanced by derivatisation, which provides a basis for further study of polysaccharides and their derivatives, and also lays a good experimental foundation for further exploration and application of polysaccharides.

**Figure 4.**
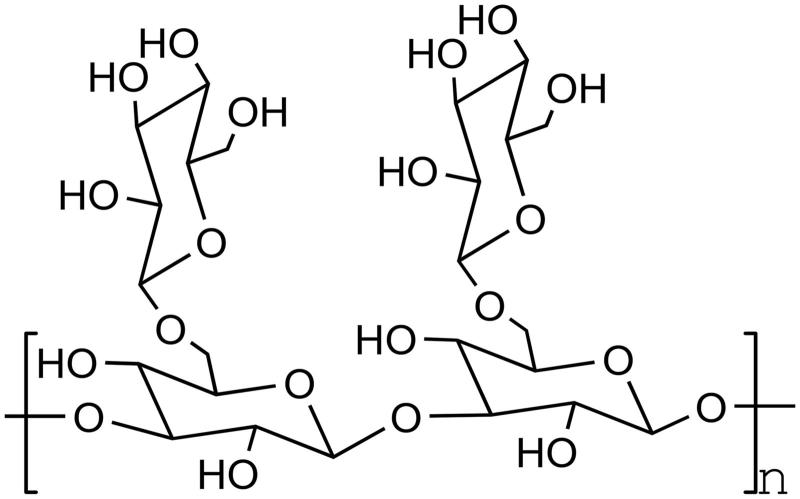
The structure of lentinan.

**Figure 5.**
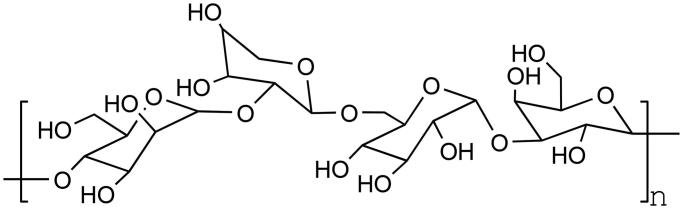
The structure of *Tremella* polysaccharide.

**Figure 6.**
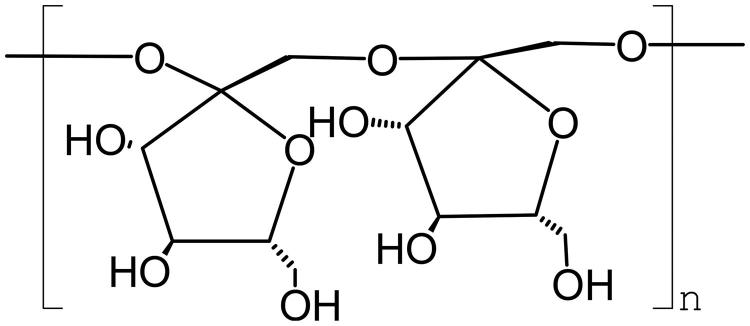
The structure of *Codonopsis* polysaccharide.

**Figure 7.**
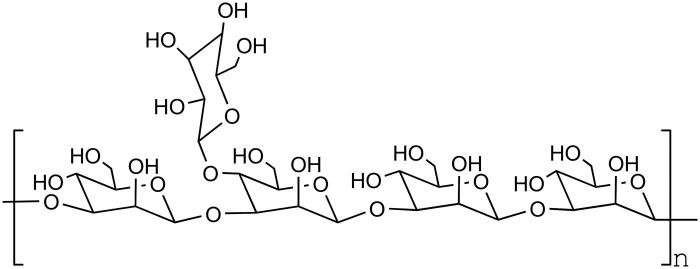
The structure of *Konjac* polysaccharide.

The structures of lentinus edodes polysaccharide, tremella polysaccharide, radix codonopsis polysaccharide, and konjac glucomannan are as follows (Figures 4–7).

## 4. Conclusion

Water extraction and alcohol precipitation method has the advantages of low cost, nontoxic, and safe operation, but it has the disadvantages of long extraction time and low extraction rate. The extraction rate of dilute alkali is higher than that of water extraction and alcohol precipitation, but the extraction conditions are harsh, which can easily destroy the three-dimensional structure of polysaccharide and affect the biological activity of traditional Chinese medicine polysaccharide. Therefore, it is mainly applied to the extraction of alkali-soluble polysaccharides. Liquid fermentation extraction method has the advantages of high yield, short cycle, simple operation, resource saving, and large industrial production scale, and has gradually become the main method to obtain polysaccharides. Fermentation is carried out in production equipment. Enzymatic extraction by enzymatic degradation of cell wall to promote the polysaccharide extraction, this method can greatly improve the extraction yield of water-soluble polysaccharide, method is simple, but the strict temperature, pH, temperature, temperature, temperature, temperature, temperature, temperature, temperature, temperature, temperature, temperature, temperature, temperature, temperature, temperature, temperature, temperature, humidity, etc.

*Reaction time and enzyme concentration conditions*. Higher temperature will lead to the deactivation of enzymes, so the extraction rate is lower, followed by the number of enzymes. With the increase of enzyme dosage, the cell wall of plant is hydrolysed by enzyme, which accelerates the dissolution of polysaccharide. However, after consumption to a certain extent, the enzyme decomposition of Chinese traditional medicine polysaccharide intensifies, the yield decreases, and it is the digestion time. With the prolongation of digestion time, the barrier layer formed by cellulose and pectin is effectively removed. When certain enzymatic hydrolysis time is reached, the effect of further prolongation time is not obvious, and the yield of Chinese traditional medicine polysaccharide will be significantly reduced. Using the energy of microwave radiation and ultrasonic radiation can quickly destroy the cell wall, so as to quickly extract polysaccharide, with high extraction rate, simple operation, fast speed, good selectivity, less solvent, and simple operation. Vacuum extraction technology has the advantages of high extraction rate, low extraction temperature, low damage to temperature-sensitive components, short extraction time, less macromolecular impurities, extraction liquid clarification, low energy consumption, and combines microwave technology with traditional extraction technology. E technology, ultrasonic extraction technology, enzyme technology, and traditional water technology provide new ideas and methods for the extraction of traditional Chinese medicine polysaccharide, the extraction process will be more mature, and will be widely used in the industry of traditional Chinese medicine extraction.

*Chinese medicine polysaccharide*. Oxidation mechanism is to directly scavenge free radicals, inhibit the formation of free radicals, and activate the antioxidant system. Some antioxidants are free radical scavengers that bind to intermediates in the oxidation process, making oxidation impossible. They release hydrogen ions and break down peroxides during oxidation, so oxidation cannot continue, and some antioxidants, such as scavenging free radicals from superoxides, block or weaken oxidase activity. Some can be used to reduce the ability of metal ions to chelate through metal ions to promote oxidation. Some polysaccharides have antioxidant activity, while others do not. However, some traditional Chinese medicine polysaccharides have antioxidant activities, which may be due to the influence of other components of some traditional Chinese medicine, so it is very important to find out their specific antioxidant activities. The composition and content of Chinese traditional medicine polysaccharide, because the antioxidant capacity of polysaccharide generally increases with the increase of concentration, that is, the higher the concentration, the higher the antioxidant capacity. Different polysaccharides have different antioxidant activities. Here is the spatial structure of polysaccharides. Some traditional Chinese medicine polysaccharides have the same monosaccharide composition, but due to their different spatial structure, they have different antioxidant activities. In addition, polysaccharides from the same Chinese medicine have different activities because they have different activities. Different extraction methods can obtain polysaccharides with different purity, structure or molecular weight. Therefore, the internal factors affecting the antioxidant activity of Chinese traditional medicine polysaccharides are the types and spatial structure of polysaccharides. The main factors influencing the spatial structure of Chinese traditional medicine polysaccharides are the source, growth period, preparation method, and drying method. In order to obtain antioxidant activity, it is necessary to select the appropriate Chinese medicine at the best time, extract polysaccharide in the best way and preserve it under the best conditions.

In summary, the antioxidant activity of Chinese traditional medicine polysaccharides has an important relationship with its first-order structure, advanced structure, molecular weight, solubility, etc., but it lacks scientific basis and regular conclusion, relying mainly on speculation and analysis. *Analysis*. Therefore, it is limited to elucidate the antioxidant mechanism of TCM polysaccharides at the molecular level, which needs to be further studied.
